# Supplementary material for: Neural representation of time across complementary reference frames
Source: eLife. 2026 May 8;14:RP107273. doi: 10.7554/eLife.107273 (PMC13155752; doi:10.7554/eLife.107273)
Supplement: Supplementary file 1. [file elife-107273-supp1.pdf]

Supplementary File 1: The reaction time of the corrected trials indicates that time is differently processed under internal and external perspectives[1]

Fixed Effects[2]	df	F	p
Sequential Distance	1, 6914	0.018	0.895
Duration	1, 6914	7.687	0.006
Same vs. Different	1, 6914	0.862	0.353
Future vs. Past	1, 6914	0.088	0.766
Syllable Length	1, 6914	33.222	< 0.001
Task Type	1, 6914	5.845	0.016
Task Type × Sequential Distance	1, 6914	13.959	< 0.001
Task Type × Duration	1, 6914	6.246	0.012
Task Type × (Same vs. Different)	1, 6914	70.490	< 0.001
Task Type × (Future vs. Past)	1, 6914	0.002	0.965
Task Type × Syllable Length	1, 6914	0.961	0.327

[1] Linear Mixed Model: RT ~ 1 + Task Type * (Sequential Distance + Duration + Same/Different + Future/Past + Syllable Length) + (1 | Participant)

[2] The significant effects were highlighted. We did not highlight the significant main effects if the corresponding interaction effects were also significant.
